# Leveraging Existing Birth Defects Surveillance Infrastructure to Build Neonatal Abstinence Syndrome Surveillance Systems — Illinois, New Mexico, and Vermont, 2015–2016

**DOI:** 10.15585/mmwr.mm6807a3

**Published:** 2019-02-22

**Authors:** Jennifer N. Lind, Elizabeth C. Ailes, Caroline C. Alter, Jane E. Fornoff, Peggy Brozicevic, Luigi F. Garcia Saavedra, Laura E. Tomedi, Melissa Gambatese, Barbara Carroll, Lucia Orantes, Brennan Martin, Ashley A. Horne, Jennita Reefhuis

**Affiliations:** ^1^National Center on Birth Defects and Developmental Disabilities, CDC; ^2^March of Dimes, White Plains, New York; ^3^Illinois Department of Public Health; ^4^Vermont Department of Health; ^5^New Mexico Department of Health.

Neonatal abstinence syndrome (NAS) is a drug withdrawal syndrome that can occur following prenatal exposure to opioids (*1*). NAS surveillance in the United States is based largely on diagnosis codes in hospital discharge data, without validation of these codes or case confirmation. During 2004–2014, reported NAS incidence increased from 1.5 to 8.0 per 1,000 U.S. hospital births (*2*), based on *International Classification of Diseases, Ninth Revision, Clinical Modification* (ICD-9-CM) diagnosis codes identified in hospital discharge data, without case confirmation. However, little is known about how well these codes identify NAS or how the October 1, 2015, transition from ICD-9-CM to the tenth revision of ICD-CM (ICD-10-CM) codes affected estimated NAS incidence. This report describes a pilot project in Illinois, New Mexico, and Vermont to use birth defects surveillance infrastructure to obtain state-level, population-based estimates of NAS incidence among births in 2015 (all three states) and 2016 (Illinois) using hospital discharge records and other sources (varied by state) with case confirmation, and to evaluate the validity of NAS diagnosis codes used by each state. Wide variation in NAS incidence was observed across the three states. In 2015, NAS incidence for Illinois, New Mexico, and Vermont was 3.0, 7.5, and 30.8 per 1,000 births, respectively. Among evaluated diagnosis codes, those with the highest positive predictive values (PPVs) for identifying confirmed cases of NAS, based on a uniform case definition, were drug withdrawal syndrome in a newborn (ICD-9-CM code 779.5; state range = 58.6%–80.2%) and drug withdrawal, infant of dependent mother (ICD-10-CM code P96.1; state range = 58.5%–80.2%). The methods used to assess NAS incidence in this pilot project might help inform other states’ NAS surveillance efforts.

Through a competitive application process, the March of Dimes, a nonprofit that works to improve the health of mothers and their babies (https://www.marchofdimes.org), in collaboration with CDC, awarded grants to CDC-funded, state-based birth defects programs in Illinois, New Mexico, and Vermont to adapt birth defects surveillance methodology to conduct active, population-based surveillance for NAS or passive case-finding with case confirmation. Each state defined a population-based 2015 birth cohort in which to identify infants with NAS; Illinois extended data collection to include a 2016 birth cohort. All three states used hospital discharge data to identify potential cases using infant ICD-9-CM diagnosis codes 779.5 (drug withdrawal syndrome in a newborn) and 760.72 (noxious influences affecting fetus or newborn via placenta or breast milk, narcotics) and ICD-10-CM codes P96.1 (drug withdrawal, infant of dependent mother) and P04.49 (newborn affected by maternal use of other drugs of addiction), as well as other infant and maternal diagnosis codes of interest to the state. Illinois used two additional data sources: 1) the Illinois birth defects registry’s Adverse Pregnancy Outcomes Reporting System,* which collects information on infants born to Illinois residents with documented prenatal opioid exposure or withdrawal symptoms during their newborn hospitalizations; and 2) reports of infants with NAS scores >8 (typically on a scale of 0–37) from selected hospitals (*3*). Vermont also queried Medicaid claims data and Vermont’s all-payer claims database, the Green Mountain Care Board’s Vermont Health Care Uniform Reporting and Evaluation System (https://gmcboard.vermont.gov/health-data-resources/vhcures), for commercial claims data, as a part of a Birth Information Network established for surveillance for birth defects and other congenital conditions. In all three states, potential cases were identified among the 2015–2016 birth cohorts and then deduplicated.

States abstracted all available infant and maternal medical records of identified potential NAS cases to confirm the diagnosis.^†^ A uniform clinical case definition, which expands on a previously published case definition (*4*), was then applied to potential cases (Box). The overall state-level confirmed population-based NAS incidence per 1,000 births (from all available data sources) and confirmed NAS incidence by data source were calculated. PPV was calculated by diagnosis code in medical records, defined as the number of confirmed NAS cases divided by the total number of potential NAS cases identified, multiplied by 100.

BOX Clinical case definition used to confirm cases of neonatal abstinence syndrome (NAS)* — Illinois, New Mexico, and Vermont, 2015 and Illinois, 2016

**To meet the NAS clinical case definition, all of the following must occur:**

1. Presence of a constellation of clinical signs consistent with NAS (i.e., a documented NAS score >8 [on a scale of 0–37]), not explained by another etiology or a documented infant diagnosis of NAS with pharmacologic treatment;
2. Documented history of maternal use during pregnancy of prescription or illicit drugs associated with NAS or laboratory confirmation of recent maternal drug use or fetal exposure to such drugs;
3. Severity of illness that resulted in a prolonged (>2 days) neonatal hospitalization.

* The original clinical case definition (https://www.cdc.gov/mmwr/preview/mmwrhtml/mm6408a3.htm) was expanded for this project to include “or a documented infant diagnosis of NAS with pharmacologic treatment” in the first criterion.

In 2015, NAS incidence was 3.0 per 1,000 births in Illinois, 7.5 in New Mexico, and 30.8 in Vermont (Table). In Illinois, data from hospital discharge data provided the highest estimate of NAS incidence (2.7 per 1,000 births) compared with data from the Adverse Pregnancy Outcomes Reporting System (2.2) and from hospitals that provided NAS scores (0.4). In Vermont, Medicaid data^§^ provided the highest estimate of NAS (62.3); incidence estimates based on hospital discharge data (29.6) and commercial claims data (1.6) were lower. The overall incidence of NAS in Illinois in 2016 remained at 3.0 per 1,000 births.

**TABLE:** Incidence of confirmed neonatal abstinence syndrome (NAS), by state and data source — Illinois, New Mexico, and Vermont, 2015 and Illinois, 2016

Data source	Illinois	New Mexico	Vermont
	No. of confirmed cases (cases per 1,000 births*)	No. of confirmed cases (cases per 1,000 births^†^)	No. of confirmed cases (cases per 1,000 births^§^)
**2015** ^¶^	**474 (3.0)**	**194 (7.5)**	**160 (30.8)**
Hospital discharge data**	433 (2.7)	194 (7.5)	154 (29.6)
Adverse Pregnancy Outcomes Reporting System	351 (2.2)	—^§§^	—^§§^
Hospital-provided NAS score	70 (0.4)	—^§§^	—^§§^
Medicaid claims	—^§§^	—^§§^	144 (62.3)
Commercial claims	—^§§^	—^§§^	—^††^ (1.6)
**2016^¶^**	**470 (3.0)**	**—^§§^**	**—^§§^**
Hospital discharge data**	442 (2.9)	—^§§^	—^§§^
Adverse Pregnancy Outcomes Reporting System	336 (2.2)	—^§§^	—^§§^
Hospital-provided NAS score	9 (0.1)	—^§§^	—^§§^
Medicaid claims	—^§§^	—^§§^	—^§§^
Commercial claims	—^§§^	—^§§^	—^§§^

* Denominator = Illinois resident live births delivered in, or transferred to, a hospital in the Illinois Perinatal Network.

^†^ Denominator = New Mexico resident births occurring in New Mexico.

^§^ Denominator = Vermont resident births occurring in Vermont from birth file. Denominators for Medicaid claims and commercial claims data defined by payer in birth file.

^¶^ Data sources were not mutually exclusive; therefore, the number of cases do not sum to the total.

** Hospital discharge data include all payer types, including Medicaid.

^††^ Numbers <11 from commercial claims data have been suppressed.

^§§^ Data not available.

In all three states, the diagnosis codes with the highest PPVs for identifying confirmed cases of NAS, based on a uniform case definition were drug withdrawal syndrome in a newborn (ICD-9-CM code 779.5; state range = 58.6%–80.2%) and drug withdrawal, infant of dependent mother (ICD-10-CM code P96.1; range = 58.5%–80.2%) (Figure).

**FIGURE:**
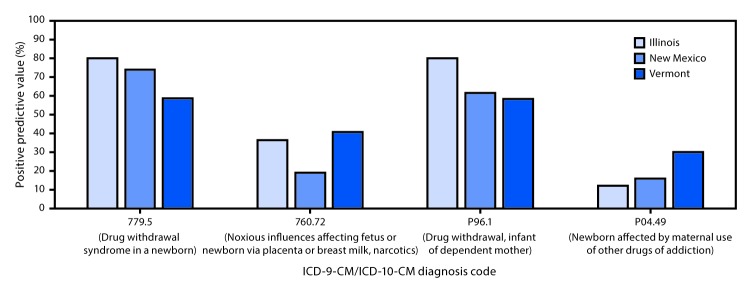
Positive predictive value* of neonatal abstinence syndrome (NAS) diagnosis codes from the ninth and tenth revisions of *International Classification of Diseases, Clinical Modification* (ICD-9-CM and ICD-10-CM), by state and infant diagnosis code^†^ — Illinois, New Mexico, and Vermont, 2015 * Positive predictive value calculated as follows: [(no. of confirmed NAS)/(no. of confirmed NAS + no. of not confirmed NAS)] x 100. ^†^ ICD-9-CM diagnosis codes 779.5 and 760.72 were used before October 1, 2015; ICD-10-CM diagnosis codes P96.1 and P04.49 became effective October 1, 2015.

## Discussion

No standardized way to conduct state-based NAS surveillance in the United States exists, and there is no standardized national surveillance system; most published NAS estimates are based on ICD-9-CM or ICD-10-CM diagnosis codes from hospital discharge data, without case confirmation, and few studies have validated these diagnosis codes for NAS surveillance (*5*,*6*). CDC has been supporting population-based surveillance for birth defects since 1967 to monitor the prevalence of birth defects and provide early warning of increases over time (*7*). In this pilot project, birth defects programs in Illinois, New Mexico, and Vermont demonstrated the feasibility of using existing birth defects surveillance methods and multiple data sources to obtain population-based estimates of NAS.

The wide variation in NAS incidence identified among the three states is consistent with the variation in state-specific prevalence of maternal opioid use disorder documented at delivery hospitalization (14.8 and 48.6 per 1,000 delivery hospitalizations in New Mexico and Vermont in 2014, respectively; data not available for Illinois) (*8*). Throughout 15 years of perinatal quality improvement, Vermont has been training personnel at birthing hospitals in the diagnosis and treatment of NAS, as well as in improving opioid agonist treatment capacity. Higher case ascertainment in states with enhanced procedures for identifying mothers with opioid use disorder and infants with NAS might, in turn, result in a higher NAS incidence.

Historically, NAS surveillance in Illinois has been conducted passively through hospital reports to the Adverse Pregnancy Outcomes Reporting System, and no published estimates in the literature of state-level NAS incidence currently exist. This report found that Illinois’ passive surveillance methods, based on the Adverse Pregnancy Outcomes Reporting System (2.2 per 1,000 births), might underestimate the incidence of NAS. Illinois’ NAS incidence from this report was lower than a published hospital discharge data–based regional estimate (6.9 per 1,000 births in Illinois, Indiana, Michigan, Ohio, and Wisconsin, in 2012) but similar to a 2013 estimate in Iowa (2.2) (*9*,*10*). Previous hospital discharge data–based estimates, without case confirmation, for New Mexico and Vermont were 8.5 and 33.3 per 1,000 births, respectively (*10*). These estimates are slightly higher than the estimates of confirmed NAS found in this report, suggesting that hospital discharge data, without case confirmation by medical record abstraction, might slightly overestimate the prevalence of NAS.

Among the diagnosis codes evaluated, infant drug withdrawal codes (ICD-9-CM code 779.5 and ICD-10-CM code P96.1) resulted in the highest PPVs. ICD-9-CM code 779.5 has been the most commonly used infant drug withdrawal code in the United States and is the most specific ICD-9-CM code for NAS (*2*,*9*,*10*). No change in PPV after transition from ICD-9-CM code 779.5 to ICD-10-CM code P96.1 was observed in two of the three states, providing a better understanding of how the transition might affect surveillance for NAS over time. A study of Tennessee Medicaid claims data reported a PPV of 91% for the ICD-9-CM drug withdrawal code (779.5) among 950 potential NAS cases during 2009–2011 and a PPV of 98.2% for the ICD-10-CM drug withdrawal code (P96.1) among 217 potential cases during 2016 (*6*). However, the higher PPVs from that study might result from the authors’ use of a lower NAS threshold (NAS score >4) than that which was used in the present study (NAS score >8). The variation in PPVs observed across states in this study might be caused by variability in coding and case definitions across states, hospitals, and providers. Hence, a careful evaluation of the use of NAS-related diagnosis codes in a particular state is important before relying on those codes for NAS surveillance.

The findings in this report are subject to at least three limitations. First, because this analysis was restricted to data from only three states, the findings are not necessarily generalizable to the rest of the United States. Second, the sensitivity of these diagnosis codes in identifying NAS cases could not be evaluated in this report because the actual frequency of NAS in each state is unknown. Finally, the case definition required a neonatal hospitalization of >2 days; therefore, infants discharged sooner would not meet the criteria. However, this would likely apply to only a small proportion of infants because the mean length of stay for infants with NAS has been found to range from 14.9 to 16.6 days (*2*).

NAS surveillance based solely on diagnosis codes in hospital discharge data without case confirmation by medical record abstraction might slightly overestimate NAS incidence. This report provides more current, confirmed state-level, population-based estimates of NAS incidence in Illinois, New Mexico, and Vermont; demonstrates the feasibility of building on the experience of birth defects surveillance to conduct statewide NAS surveillance; and evaluates the use of diagnosis codes for identifying NAS cases. The lessons learned from this pilot project might help inform NAS surveillance efforts in other U.S. states or jurisdictions.

**Summary**

**What is already known about this topic?**

Neonatal abstinence syndrome (NAS) surveillance in the United States is based largely on diagnosis codes in hospital discharge data, without validation of these codes or case confirmation.

**What is added by this report?**

Estimates of NAS incidence during 2015 were 3.0 per 1,000 births for Illinois, 7.5 for New Mexico, and 30.8 for Vermont. Of the four diagnosis codes evaluated, those for infant drug withdrawal (779.5 and P96.1) had the highest positive predictive values for identifying confirmed NAS cases.

**What are the implications for public health practice?**

NAS surveillance based solely on diagnosis codes in hospital discharge data might slightly overestimate NAS incidence. These findings could help inform NAS surveillance in other states.

## Footnotes

* http://www.dph.illinois.gov/data-statistics/epidemiology/apors.

† Medical records of <2% of all potential cases were unable to be obtained for abstraction for reasons including that 1) the infant was born at an out of state hospital or 2) the infant was transferred out of state for treatment after birth.
